# Eczema Cured by Vaccination

**Published:** 1884-02

**Authors:** McCaul Anderson


### ECZEMA CURED BY VACCINATION.

The local inflammatory action and febrile disturbance set up by vaccination is calculated to call often forth an eruption of eczema in those who are so predisposed, and to aggravate existing attacks; so much so, indeed, that the operation is frequently delayed for many months on this account. And yet it must be admitted that in some chronic and inveterate cases it has precisely the opposite effect, and may therefore be ranked as a curative agent. As an illustration of this, two cases reported by Mr. Lawson Tait * may be mentioned. "The first," he says, "was the child of a commercial gentleman of great intelligence, who allowed me to try vaccination after everything else had been done that could be suggested. It was a most obstinate case of eczema over the whole body, the scalp being the seat of its worst display. The glands of the neck were chronically enlarged, and at one time suppurated so seriously as to endanger the child's life. Temporary benefit was derived from change of air, but drugs had no effect. Acting on the usual rule, I put off the vaccination of the child for three several periods of nine months. * * * I told the father that * * * I believed vaccination might cure the child by exercising some influence on its nutrition. He agreed to the experiment; and to diminish risk as far as possible, I used lymph which had passed through one healthy child from the heifer. The result was most remarkable, for in a few days a marked improvement was visible in the child; and in little more than three weeks all traces of the eruption had disappeared, save a roughness of the skin, which still exists. The hair grew rapidly on the scalp, and the child now is in all respects as fine an infant as I have ever seen.

"At the same time I had under my care the child of a clergyman, for which many prolonged and various courses of treatment had been adopted ineffectually for an eczematous eruption affecting the whole body, but mainly the face and flexures of the joints. It was nearly two years old, and had never been vaccinated. I told the father of the case I have just related, and he consented to the vaccination. He had sent me the following note of the history: 'To the best of my recollection, the symptoms of skin disease in baby were first manifested when she was about two months old. The disease appeared in a virulent form for the space of nine months, at the end of which time she was vaccinated. After vaccination the child improved rapidly, and

---

* *British Medical Journal*, January 27th, 1882, p. 92.

in a month not a trace of the malady was left.'" Of course, it is only in exceptional cases that such happy results can be expected.—*Dr. McCaul Anderson, in Journal of Cutaneous and Venereal Diseases.*

